# Differences in the Autophagy Response to Hypoxia in the Hippocampus and Neocortex of Rats

**DOI:** 10.3390/ijms23148002

**Published:** 2022-07-20

**Authors:** Anna Churilova, Tatiana Zachepilo, Ksenia Baranova, Elena Rybnikova

**Affiliations:** 1 Laboratory of Regulation of Brain Neuron Functions, Pavlov Institute of Physiology RAS, 199034 Saint-Petersburg, Russia; churilovaav@infran.ru (A.C.); baranovaka@infran.ru (K.B.); 2 Laboratory of Genetics of Higher Nervous Activity, Pavlov Institute of Physiology RAS, 199034 Saint-Petersburg, Russia; zachepilo_t@infran.ru

**Keywords:** autophagy, neurons, hypoxia, hippocampus, neocortex

## Abstract

Autophagy is a regulated mechanism of degradation of misfolded proteins and organelles in the cell. Neurons are highly differentiated cells with extended projections, and therefore, their functioning largely depends on the mechanisms of autophagy. For the first time in an animal model using immunohistochemistry, dot analysis, and qRT-PCR, the autophagy (macroautophagy) activity in neurons of two brain regions (hippocampus and neocortex) under normoxia and after exposure to hypoxia was studied. It was found that under normoxia, the autophagic activity was higher in the hippocampal neurons than in the neocortex of rats. In the hippocampus, the exposure of rats to hypoxia resulted in a decrease in the content of autophagy markers LC3 and p62, which was followed by activation of the autophagy-related gene expression. In the neocortex, no changes in these marker proteins were observed after the exposure to hypoxia. These data indicate that the neurons in the hippocampus and neocortex differ in the autophagy response to hypoxia, which may reflect the physiological and functional differences of the pyramidal cells of these brain regions and may to some extent account for the extreme vulnerability of the CA1 hippocampal neurons and relatively high resistance of the neocortical neurons to hypoxia.

## 1. Introduction

Autophagy is a regulated lysosomal-dependent process of recycling and degradation of misfolded proteins and organelles in the cell [1,2,3]. There are several major variants of autophagy: chaperon-mediated autophagy, macroautophagy and microautophagy. In macroautophagy (here and onwards referred to as autophagy), the protein aggregates and the whole cellular organelles such as mitochondria, endoplasmic reticulum, and ribosomes are sequestered into the double-membrane structures (autophagosome), which then fuse with the lysosomes. All steps of this process, i.e., receptor recognition, cargo delivery, autophagosome formation and fusion with the lysosome, are tightly regulated. The functional significance of autophagy is to isolate the toxic components produced by the cell during its lifespan and to provide the energy source for new biosynthesis from the products of autophagic recycling. Thus, autophagy represents a uniform mechanism for maintaining cellular homeostasis, which is important both under the resting and cellular stress conditions.

The autophagy mechanism becomes extremely important for post-mitotic cells, as they cannot eliminate the toxic cargo by cell division and thus need a special mechanism of autophagy for utilizing it. Neurons are highly differentiated cells with a unique structure holding extremely long axons and branched dendrites and with a high level of functional activity. Bearing such long projections represents a challenge for neuronal cells, as their functionality has to be maintained all the time at a great distance from the soma. It is known that proteins and organelles from the distal parts of axon, dendrites and even postsynaptic membranes are transported via autophagosomes to the soma of the cell, where they fuse with the lysosome directing the cargo for degradation [4]. Therefore, neurons are highly dependent on the mechanisms of autophagy [4,5]. Indeed, there is evidence that mutations in autophagy-related genes are associated with neuronal loss and neurodegeneration [6,7,8]. Impairments in the autophagy pathway are known to contribute to the progression of such neurodegenerative diseases as lateral sclerosis, Huntington’s, Parkinson’s, and Alzheimer’s diseases [9,10,11,12]. Thus, the importance of the autophagic mechanism for neuronal functioning makes it an attractive target for investigation and the components of the autophagy cascade—the potential targets for medical treatment.

It is known that hypoxia stimulates autophagy in vivo, but the effects of autophagy activation on neuronal functions during hypoxia are contradictory. In some cases, the activation of autophagy is associated with cell survival [13,14,15,16,17], whereas there is also evidence that autophagy together with apoptosis can contribute to cell death [18,19,20,21]. Using a model of hypobaric hypoxia in rat, which is well established in our laboratory, we have demonstrated that severe hypoxia causes memory and behavioral impairments and functional disorders in animals during the first days after the exposure [22,23]. The hippocampus is known to play an important role in learning and memory as well as regulation of the hypothalamic–pituitary–adrenal axis [24]. The fronto-parietal neocortex containing large pyramidal neurons is a main region of integration and processing of somatosensory information, which makes this area responsible for the regulation of reaction of the organism to different external stimuli. In the previous studies, the antioxidant, transcription factors and neurotrophin expression have been investigated in the hippocampus and neocortex of rats after hypoxia with an emphasis on the fact that the hippocampus was more vulnerable to hypoxia challenge [25,26,27]. As soon as autophagy is a mechanism promoting cell survival [13], it may play an important role in coping with stress to hypoxia and may at least in part explain the different vulnerability of pyramidal neurons of the hippocampus and neocortex to hypoxia. However, how autophagy is affected by hypobaric hypoxia in pyramidal neurons in different brain regions is still to be elucidated. As such, the aim of this study was to compare the autophagy process in two brain regions: the hippocampus, which relates to the archicortex, and the neocortex of rats under normoxia and after exposure to hypoxia.

## 2. Results

### 2.1. Assessment of Autophagic Flux in the Hippocampus and Neocortex of Rats under Normoxia

Autophagy is a dynamic process characterized by permanent autophagosome formation, its fusion with lysosomes and cargo degradation. The rate of autophagosome and hence the autophagy protein markers turnover reflects the degradation activity of the autophagic process and is termed autophagic flux. One of the approaches to assess the autophagic flux is to estimate the accumulation of the autophagosomes or autophagy protein markers in a given time period after the application of an autophagy inhibitor, which blocks the lysosomal degradation [28,29,30]. Chloroquine (CQ) is a lysosomal inhibitor that acts by enhancing the pH of lysosomes, leading to a block in lysosomal degradation. Although there are some cautions and limitations in using CQ in vivo [31], it is the only known inhibitor to date that can be applied in vivo for assessment of autophagic flux. To assess autophagic flux, we estimated the accumulation of autophagy markers LC3 and p62 3 h, 24 h and 3 days after CQ injection. LC3 (microtubule-associated proteins 1A and 1B, MAP1LC3) is an autophagy marker that resides free in the cytosol (LC3-I form) or can be integrated into the autophagosome membrane when it becomes phosphatidylethanolamine-conjugated (LC3-II form) [32]. LC3 is important for autophagosome formation and expansion. p62/SQSMT (Sequestosome 1, ubiquitin-binding protein p62) is an adaptor protein that binds the ubiquitinated proteins with its one end and LC3-II with another, thus playing a role in tagging the cargo and its translocation inside the autophagosome. Both LC3 and p62 are known to be degraded in the lysosome with the cargo.

#### 2.1.1. LC3

**Figure 1:**
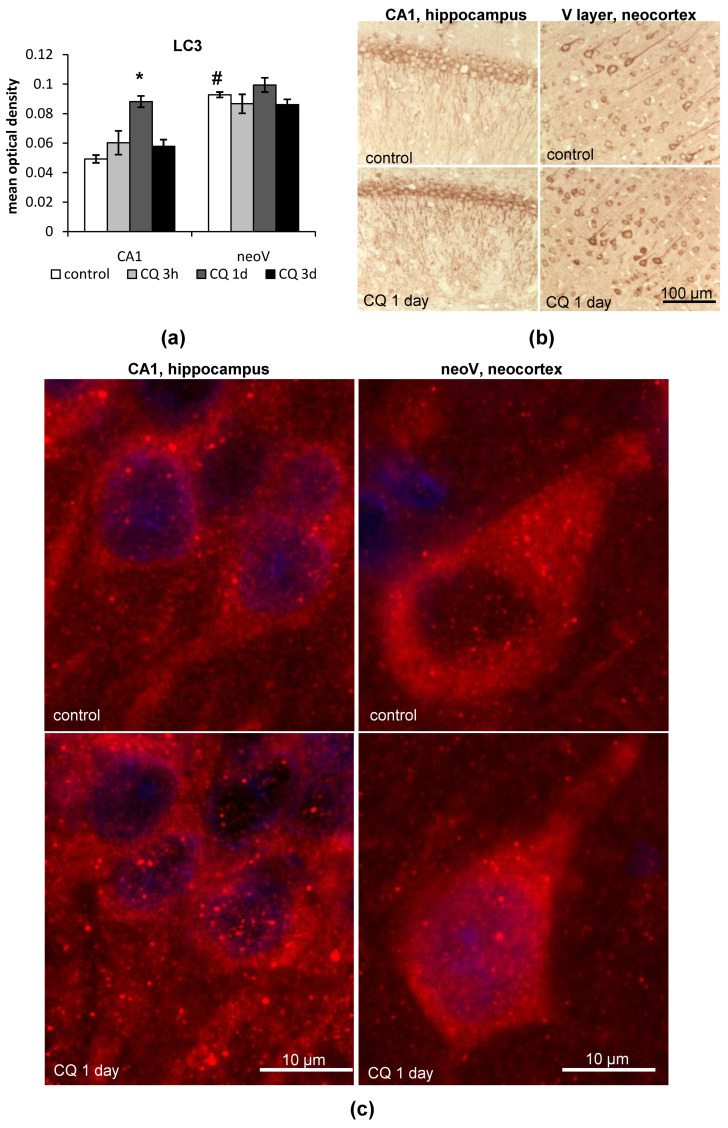
LC3 accumulation in the CA1 field of the hippocampus and the V layer of the neocortex of rats after chloroquine (CQ) injection. (**a**) Results of immunohistochemistry quantitation of LC3; (**b**,**c**) representative images of LC3 accumulation and puncta staining in the CA1 field of the hippocampus and the V layer of the neocortex of rats 1 day after CQ injections. The results are presented as the mean ± standard error of the mean. * the difference between the experimental groups within a brain region is statistically significant, *p* < 0.05; # the difference between the control groups of the hippocampus and neocortex of rats is statistically significant, *p* < 0.05.

On the whole, we have found that in control rats, the immunoreactivity to LC3 was significantly higher in neurons of the V layer of the neocortex, especially in the large pyramidal neurons, than in neurons of the CA1 field of the hippocampus (*p* = 0.0001) (Figure 1a,b). One day after CQ injection, LC3 levels were upregulated by 1.8 times in comparison to the control values in the soma of neurons of the CA1 field of the hippocampus (the CQ/control ratio = 1.796, *p* = 0.0002) (Figure 1a). Alongside this, there was also a strong accumulation of LC3 in the dendrites of the CA1 neurons of the hippocampus of rats treated with CQ (Figure 1b). However, by the third day after CQ treatment, LC3 levels did not differ from the control values in the CA1 field of the hippocampus. In the neocortex, there was a slight upregulation of LC3 levels 1 day after CQ treatment, but it was not statistically significant (Figure 1a,b).

At a higher magnification, multiple LC3-immunopositive dots, representing autophagosomes, were distinguishable on the background of the cytosolic LC3 staining both in the pyramidal neurons of the CA1 field of the hippocampus and the large pyramidal neurons of the V layer of the neocortex (Figure 1c). Both the soma of these cells and the proximal ends of the dendrites contained a significant amount of the LC3 dots. We also observed LC3 dots in neurons of the CA3, CA4 fields and dentate gyrus of the hippocampus (photos not presented). It should be mentioned that such puncta staining was also present outside the contour of the cells—in the neuropile region of the hippocampus and in the area between the neurons of the neocortex. We hypothesize that these dots represent autophagosomes located in the distal parts of the dendrites. The high concentration of LC3 in the autophagosomes is detected as a bright dot, while the contours of the dendrites cannot be revealed by LC3-immunostaining. It is hard to estimate whether the number of the dots has changed after the CQ treatment, but the intensity of the dots increased in the neurons of the CA1 field of the hippocampus (Figure 1c). In neurons of the V layer of the neocortex, the CQ treatment did not seem to affect the size or intensity of LC3 dots.

#### 2.1.2. p62

**Figure 2:**
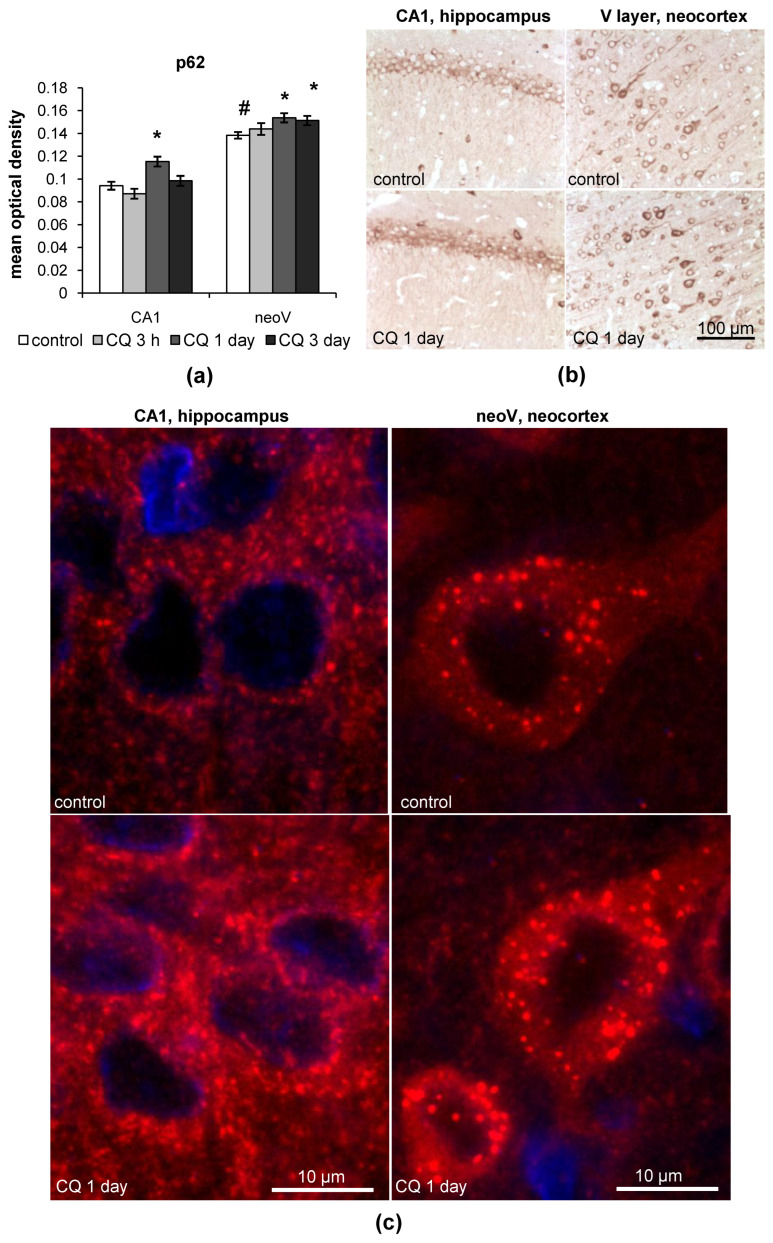
p62 accumulation in the CA1 field of the hippocampus and the V layer of the neocortex of rats after CQ injection. (**a**) Results of immunohistochemistry quantification of p62; (**b**,**c**) representative images of p62 accumulation and puncta staining in the CA1 field of the hippocampus and the V layer of the neocortex of rats 1 day after CQ injections. The results are presented as the mean ± standard error of the mean. * the difference between the experimental groups within a brain region is statistically significant, *p* < 0.05; # the difference between the control groups of the hippocampus and neocortex of rats is statistically significant, *p* < 0.05.

In control rats, p62 levels were significantly higher in neurons of the V layer of the neocortex (*p* = 0.000005), especially in the large pyramidal neurons, rather than in neurons of the CA1 field of the hippocampus (Figure 2a,b). One day after CQ injection, a slight accumulation of p62 was observed both in neurons of the CA1 field of the hippocampus (CQ/control ratio = 1.223; *p* = 0.0011) and the V layer of the neocortex (CQ/control ratio = 1.116; *p* = 0.0147) (Figure 2a,b). In the neocortex, the level of p62 was upregulated even 3 days after CQ treatment (*p* = 0.0230) (Figure 2a,b).

In the high-magnification images, it can be seen that along with p62 staining in the cytosol, there was clearly visualized puncta staining both in neurons of the hippocampus and neocortex (Figure 2c). In the CA1 field of the hippocampus, the p62 dots differed in form, while in the large pyramidal neurons of the V layer of the neocortex, the p62 dots were well-structured, intensely labeled and well distinguished from the p62 cytosolic staining. These dots were greater in size than those observed in the hippocampus and much bigger than the LC3 dots, which put a question on whether the p62 dots in neurons of the V layer of the neocortex refer to autophagosomes or probably other structures such as autophagolysosomes, lysosomes or protein conglomerates, but this is still to be clarified. Localization of the p62 dots was restricted to the soma of neurons both in the CA1 field of the hippocampus and the V layer of the neocortex. After the CQ treatment, the intensity of some of the p62 dots increased in neurons of the CA1 field of the hippocampus (Figure 2c). In the V layer of the neocortex, the number and size of the p62 dots increased after CQ treatment (Figure 2c).

### 2.2. Changes in the LC3 and p62 Levels in the Hippocampus and Neocortex of Rats after Hypobaric Hypoxia

#### 2.2.1. Immunohistochemistry

- LC3

**Figure 3:**
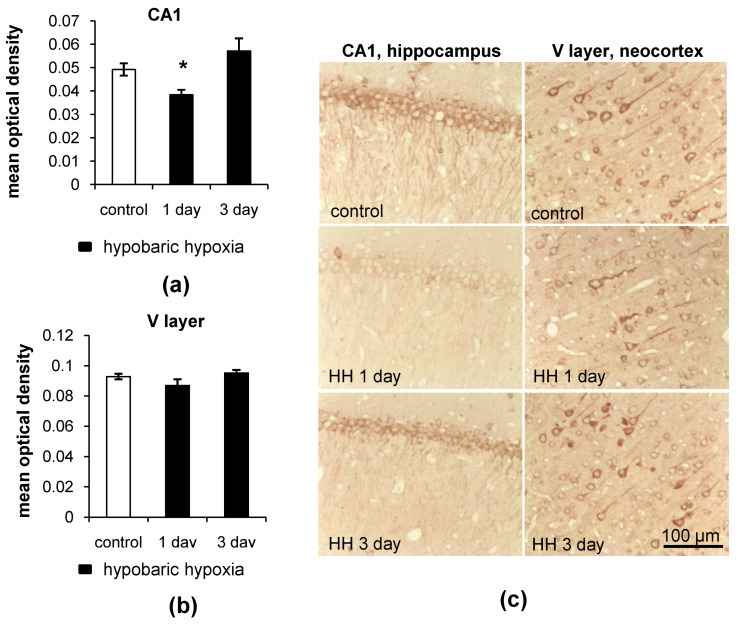
The effect of hypobaric hypoxia (HH) on LC3 protein levels in the CA1 field of the hippocampus and the V layer of the neocortex of rats. (**a**,**b**) Immunohistochemical quantification of LC3 levels; (**c**) representative images of LC3 staining. The results are presented as the mean ± standard error of the mean. * the difference between the experimental groups within a brain region is statistically significant, *p* < 0.05.

As revealed by immunohistochemical staining, hypobaric hypoxia (HH) led to a decrease in LC3 (*p* = 0.0071) levels in neurons of the CA1 field of the hippocampus of rats 1 day after the exposure compared to the control group (Figure 3a,c). However, by the third day after hypoxia, the overall LC3 levels were restored to the control values. At the same time, HH induced no changes in LC3 content in the neurons of the neocortex (Figure 3b,c).

**Figure 4:**
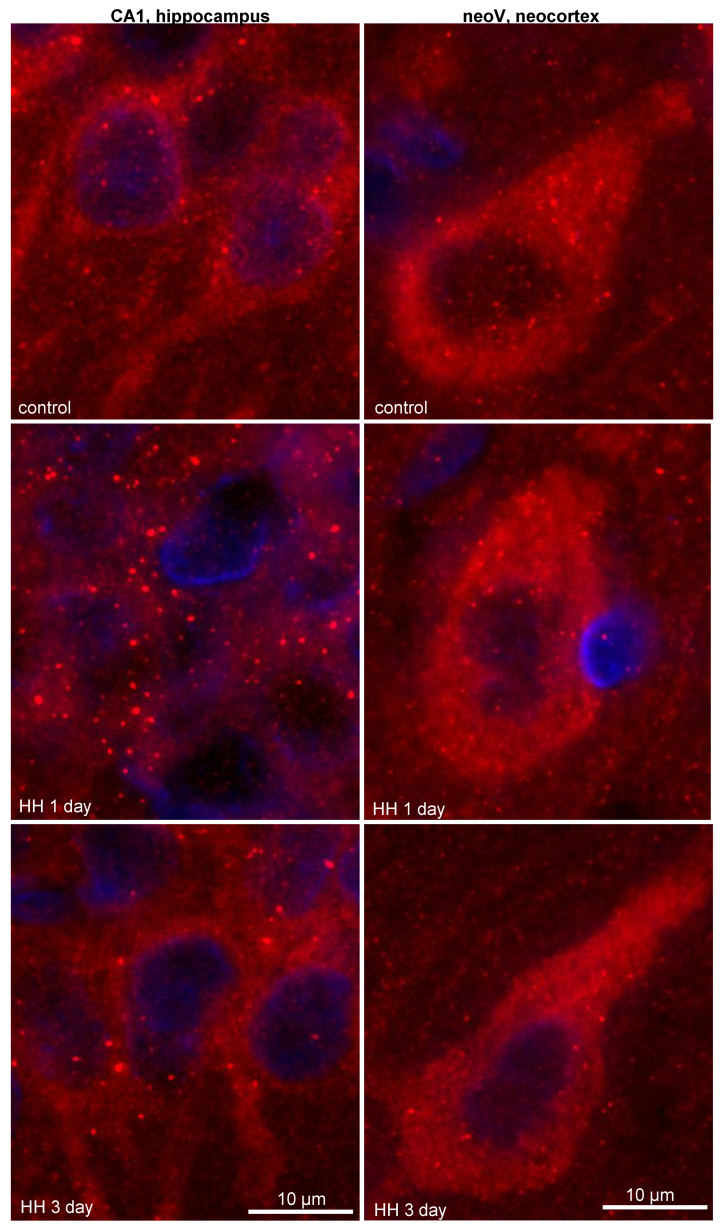
Representative images showing LC3 dots and LC3 staining in the neurons of the CA1 field of the hippocampus and the V layer of the neocortex of rats after exposure to HH.

At high-magnification analysis, it was observed that 1 day after HH, the size and intensity of the LC3 dots increased in the neurons of the CA1 field of the hippocampus (Figure 4). It is highly possible that the enlargement and increased intensity of the LC3 dots, which refer to autophagosomes, might be a result of the rapid incorporation of LC3-II into the autophagosome membrane and intensification of the autophagy. The intensification of autophagy, in turn, will lead to an increase in the consumption of LC3 and depletion of its protein pool, which is observed as a decrease in the overall LC3 content. By the third day after HH, LC3 puncta staining in the hippocampus remained unchanged but still differed from that in the control group (Figure 4). However, we did not observe any changes in the number, size or intensity of LC3 dots in the large pyramidal neurons of the V layer of rat neocortex after HH (Figure 4).

- p62

**Figure 5:**
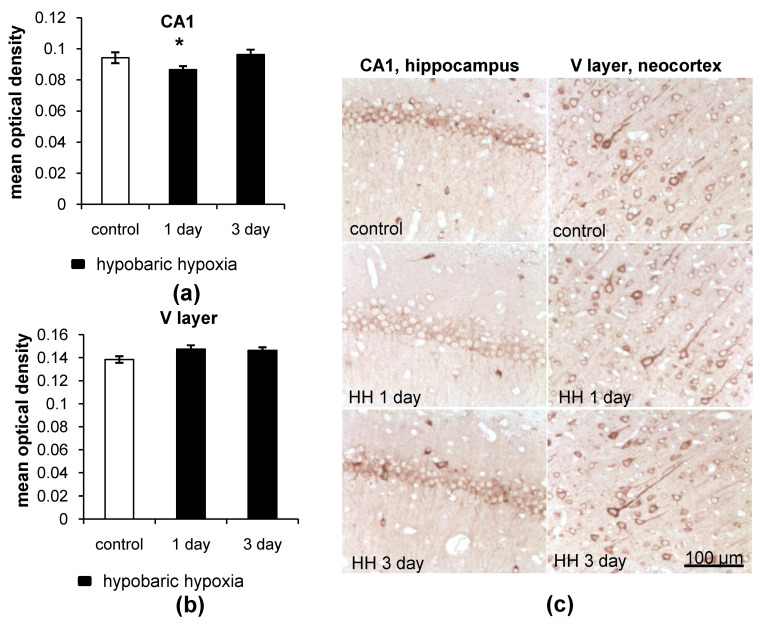
The effect of HH on the protein levels of p62 in the CA1 field of the hippocampus and the V layer of the neocortex of rats. (**a**,**b**) Immunohistochemical quantification of p62 levels; (**c**) representative images of p62 staining. The results are presented as the mean ± standard error of the mean. * the difference between the experimental groups within a brain region is statistically significant, *p* < 0.05.

**Figure 6:**
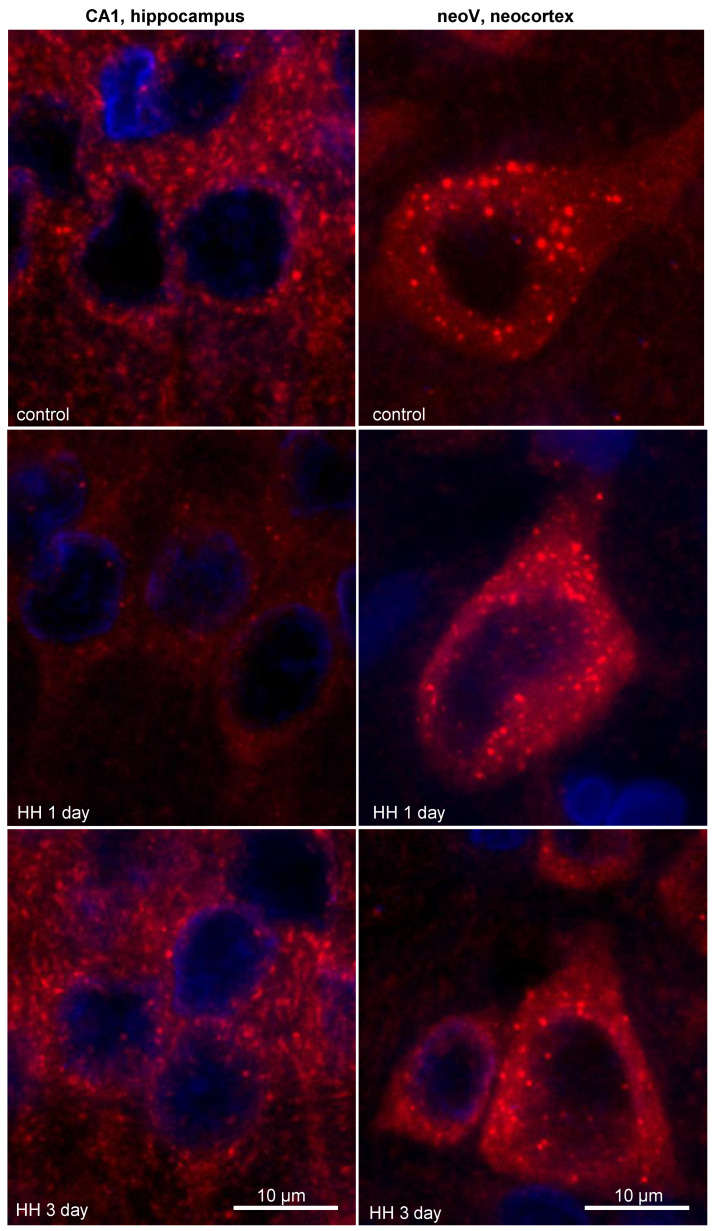
Representative images showing p62 dots and p62 staining in neurons of the CA1 field of the hippocampus and the V layer of the neocortex of rats after exposure to HH.

The levels of p62 were decreased 1 day after HH in the neurons of the CA1 field of the hippocampus (Figure 5a,c). This was also accompanied by a decrease in the intensity of the p62 puncta staining in these neurons (Figure 6). Again, we hypothesize that the intensification of autophagy could be responsible for the rapid consumption and degradation of p62 inside the lysosomes. It is well established that p62 levels are inversely correlated with autophagy activity, which means that when autophagy is activated, p62 is degraded quickly in lysosomes with its cargo [29]. Three days after HH, p62 levels and p62 puncta staining did not differ from the control values. In the neocortex, no significant changes in the p62 levels or p62 puncta staining were revealed after HH (Figure 5b,c and Figure 6).

#### 2.2.2. Dot Analysis

**Figure 7:**
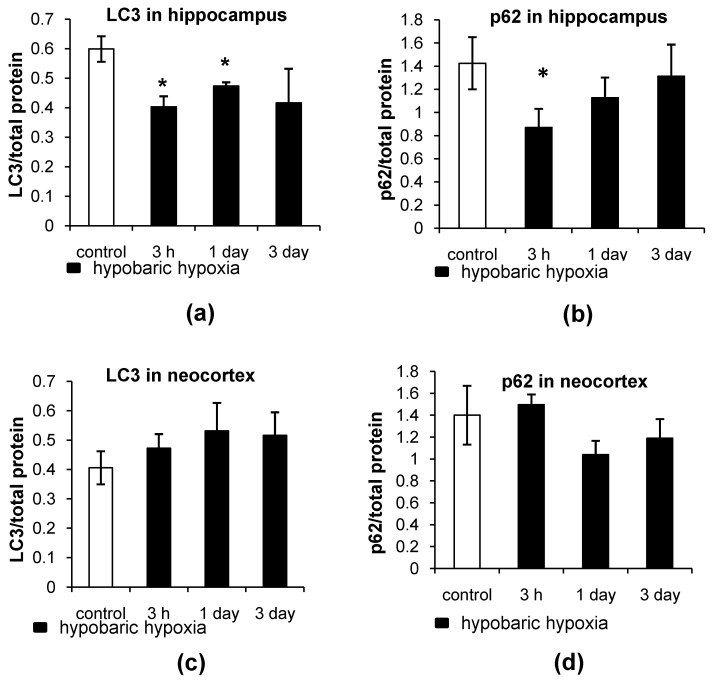
The assessment of LC3 and p62 levels in the hippocampus and neocortex of rats after exposure to HH. (**a**,**b**) Dot quantitation of LC3 and p62 levels, respectively, in the hippocampus. (**c**,**d**) Dot quantitation of LC3 and p62 levels, respectively, in the neocortex. The results are presented as the mean ± standard error of the mean. * the difference between the experimental groups within a brain region is statistically significant, *p* < 0.05.

The dot analysis has revealed that the levels of LC3 and p62 were decreased (*p* = 0.0209 and *p* = 0.0143, respectively) as early as 3 h after HH in the hippocampus of rats (Figure 7a,b). The levels of LC3 were still below the control values (*p* = 0.0339) 1 day after HH, which correlates with the immunohistochemistry results. At the same time, there was no decrease in the LC3 and p62 protein levels in the neocortex of rats (Figure 7c,d).

### 2.3. Different Patterns of Autophagy-Related Gene Expression in the Hippocampus and Neocortex of Rats in Response to Hypobaric Hypoxia

**Figure 8:**
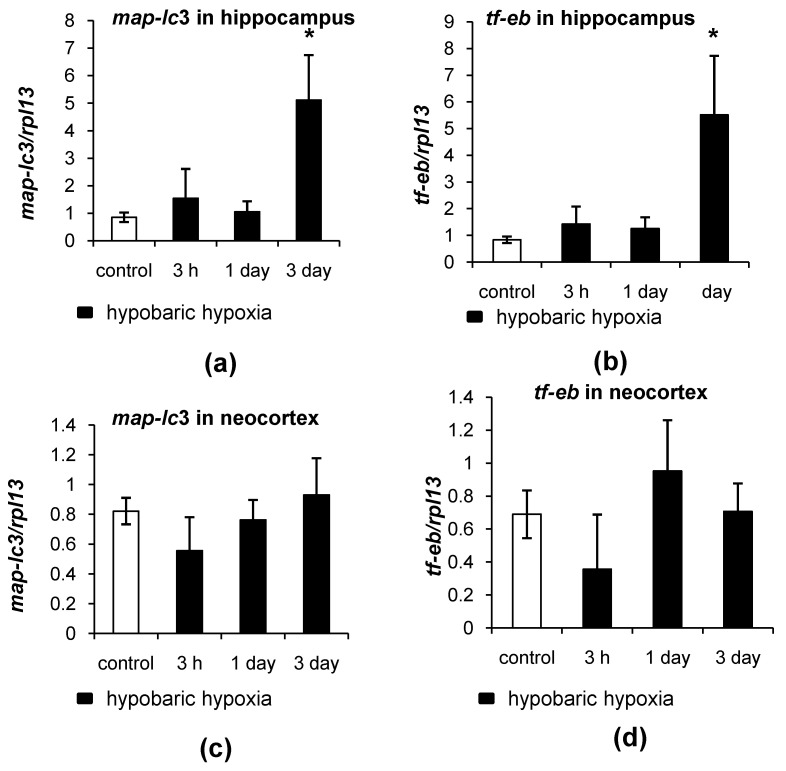
The effect of HH on the expression of autophagy-related genes in the hippocampus and neocortex of rats. (**a**,**b**) Relative mRNA levels of *map-lc3* and *tf-eb*, respectively, in the hippocampus. (**c**,**d**) Relative mRNA levels of *map-lc3* and *tf-eb*, respectively, in the neocortex. The results are presented as the mean ± standard error of the mean. * the difference between the experimental groups within a brain region is statistically significant, *p* < 0.05.

To estimate the autophagy changes after HH, the mRNA levels of autophagy-related genes *maplc3* (encoding LC3) and *tf-eb* (encoding a transcriptional factor EB that regulates the transcription of a series of autophagy-related proteins and factors of autophagosome formation) were assessed. It should be mentioned that the levels of *maplc3* expression in the hippocampus and neocortex of control rats were nearly the same (0.8521 arbitrary units in the hippocampus versus 0.8220 in the neocortex) (Figure 8a,c). The expression levels of *tf-eb* were higher in the hippocampus than in the neocortex (0.8328 versus 0.6893, respectively) (Figure 8b,d). After 3 h and 1 day after HH, there were no changes in the mRNA levels of *maplc3* and *tf-eb* in the hippocampus of rats; however, by the third day after hypoxia, their expression was significantly increased (*p* = 0.0212 and *p* = 0.0253, respectively) (Figure 8a,b). In the neocortex, no changes in the mRNA levels of *maplc3* and *tf-eb* after HH were observed (Figure 8c,d).

## 3. Discussion

A large number of studies demonstrated an extremely important role of autophagy for the homeostasis of cells [6,7] and especially for the normal functioning of neurons [4,33]. There is a growing body of evidence of the critical role that autophagy plays in the development of neuronal processes and maintenance of synaptic functions [34,35,36,37,38,39]. Still, not much attention has been dedicated to the variability that may come from the specificity of neurons of different brain regions. There is some evidence that autophagy can be differentially regulated in various brain regions under fasting, which probably depends on the specific functions in which the neurons of a particular brain region are involved [40]. In this work, we focused on the specific features of the autophagy process attributable to the neurons in the hippocampus and neocortex of rats.

In the present study, it was found that the pyramidal neurons of the CA1 field of the hippocampus and the V layer of the neocortex demonstrate different autophagy dynamics. This was evident from the differences in LC3 levels, rates of accumulation of LC3 and LC3 puncta staining after CQ injections between the hippocampus and neocortex of rats. In addition to LC3, p62 levels and *tf-eb* expression levels were also different in these brain regions. Such a difference in the autophagy may arise from the differences in the physiological functions attributed to the hippocampus and neocortex. Depending on the specificity of their functions, pyramidal neurons can vary to a great extent in dendritic structure, possessing dendritic domains with distinct synaptic inputs, excitability and modulation [41]. In particular, these features underlie the indispensable role of the hippocampus in the processes of learning and memory and determine the high level of synaptic plasticity of the hippocampal neurons. Synaptic plasticity is regulated by the quantity and composition of receptors at the postsynaptic membrane with NMDA and AMPA receptors being essential [42]. In the CA1 field of the hippocampus, the concentration of NMDA receptors is the highest in the brain [43]. It has been shown that the stimulation of NMDA receptors by long-term depression leads to autophagosome formation and directs AMPA receptors for lysosomal degradation [44]. It was proposed that by this means, autophagy (macroautophagy) may influence the synaptic plasticity, which consequently determines the processes of learning and memory. Indeed, there is strong evidence that the induction of autophagy in the hippocampus is required for learning and memory [45,46]. Alongside AMPA receptors, other proteins, both at presynaptic and postsynaptic membranes, have been shown to be degraded via autophagy [36,40]. Thus, taking into account the unique role of the hippocampus in memory and a large number of NMDA receptors in this brain structure, it is very likely that the aforementioned facts can partly explain the demand for a high level of autophagy activity in the neurons of the hippocampus, especially in the dendrites.

The difference in the autophagy dynamics between the hippocampus and neocortex becomes even more evident under hypoxic conditions. In our model of HH, it was found that hypoxia intensified autophagy in the neurons of the CA1 field of the hippocampus. Firstly, this was associated with a decrease in the protein levels of LC3 and an increase in the size and intensity of LC3 dots in neurons of the CA1 field of the hippocampus. This could be explained by the fact that when autophagy is intensified, the incorporation of LC3-II into autophagosome membrane rises. As a result, the observed increase in the intensity and size of LC3 dots could occur. At the same time, the demand of the cell for LC3 and its utilization is growing. As we have shown, mRNA levels of the *maplc3* gene were at the control levels 1 day after HH, which means that the synthesis of LC3 de novo does not take place and as such could not compensate for the increased demand of the cell in LC3 at this time frame. This could be one of the reasons why the overall LC3 protein levels were lowered 1 day after HH. The decrease in LC3 levels is often associated with an intensification of protein degradation and is thought to be a sign of autophagy activation [32]. Secondly, a decrease in the total p62 protein levels and the intensity of p62 puncta staining in the hippocampus 1 day after HH also indicated the autophagy intensification. The levels of p62 are known to be inversely correlated with the activity of autophagy [29]. It is highly probable that p62, which is an adaptor protein, could be rapidly degraded in response to the autophagy intensification caused by HH in the phagolysosomes or lysosomes, leading to fading of the p62 puncta staining. By the 3rd day after HH, the mRNA levels of *maplc3* and *tf-eb* in the hippocampus were increased. This allowed us to hypothesize that the activation of gene expression could serve as a compensative mechanism aimed at replenishing the LC3 and p62 protein pools. Indeed, by the 3rd day after HH, the levels of LC3 and p62 were restored to the control values.

At the same time, we did not observe any significant changes in the LC3 and p62 total protein levels or LC3 and p62 puncta staining in the neurons of the V layer of the neocortex after HH. Along with this, no changes in the mRNA levels of *maplc3* and *tf-eb* genes were found. These data suggest that autophagy was either not activated in the neurons of the neocortex at all, or it was activated to a lesser extent in comparison to the hippocampus. It is possible that the pool of LC3 and p62 in the neurons of the neocortex (especially the large pyramidal neurons of the V layer) was sufficient to cover the increased demand of the cells, and additional transcription of autophagy-related genes was not needed. However, there might be another mechanism involved; e.g., the increased translation of existing mRNA for protein synthesis de novo could be responsible for the stable protein levels and unchanged mRNA levels in the neurons of the neocortex. On the other hand, the neocortex is more resistant to hypoxia than the hippocampus, and thus, the changes in autophagy dynamics could be less prominent in the neocortex compared to the hippocampus. This observation is well correlated with the finding that oxidative stress and reduction in antioxidant defense are manifested in the hippocampus but are almost absent in the neocortex of rats after HH [47]. An alternative cause of different reactions of the hippocampus and neocortex to hypoxia might be the different composition of the receptors on the surface of the neurons, and, as a consequence, of the downstream intracellular pathways involved. While CA1 neurons carry a great number of NMDA receptors on their surface, many cortical neurons are enriched in GABA receptors [48]. An important component of GABA receptor turnover is GABARAP (a protein associated with the GABA receptor). However, GABARAP is a multifunctional protein, and its second function is participation in autophagy [49]. GABARAP is required for maturation of the autophagosomes, and the link between GABA signaling and autophagy has been well documented [50,51,52,53]. It is possible that the active use of GABARAP in the turnover of GABA receptors in the neurons of the neocortex can shift the balance toward chaperone-mediated autophagy. However, this suggestion needs further investigation.

### Limitations of the Study

(1) During immunohistochemistry, we analyzed the staining of pyramidal neuronal cells in the CA1 region of the hippocampus and the V layer of the neocortex. On this basis, we mean that the changes we describe are related to neurons. At the same time, during the dot analysis and qRT-PCR, extracts of tissues with different cell populations containing not only neuronal cells were used. (2) When discussing the results of immunohistochemistry, we point out specific changes in the CA1 region of the hippocampus and the V layer of the neocortex, but when performing biochemical analysis and qRT-PCR, we discuss the entire hippocampus and the frontal–parietal region of the neocortex.

## 4. Materials and Methods

### 4.1. Experimental Groups and Hypoxia Model

The experiments were carried out using male Wistar rats weighting 240–250 g. The animals were obtained from the Center for Collective Use “Biocollection of the Pavlov Institute of Physiology of the RAS”. The work with animals was conducted in accordance with Directive 2010/63/EU (https://eur-lex.europa.eu/eli/dir/2010/63/2019-06-26, accessed on 1 October 2020), was in compliance with the ARRIVE guidelines (Animal Research: Reporting of In Vivo Experiments) [54] and was approved by the ethical committee for Use of Animal Subjects of the Pavlov Institute of Physiology of RAS. All efforts were made to minimize the number of animals used and their suffering. There were three main experimental groups: (1) control group; (2) chloroquine-treated group; (3) hypobaric hypoxia group. In each experimental group, there were 5–6 animals for each time point for histological, biochemical and qRT-PCR analyses.

The severe HH corresponding to 5% of O_2_ was produced in a hypobaric chamber by maintaining the atmospheric pressure at 180 mm Hg for 3 h. This model has been designed in the laboratory and has been described in detail in our previous studies [23].

CQ, a nonselective inhibitor of autophagy, was dissolved afresh in 0.9% NaCl before use. The animals received an intraperitoneal injection of CQ in a dose of 3 mg/kg of body weight in the total volume of 200 μL of the solution for each animal. The control animals were treated with 200 μL of 0.9% NaCl, and animals subjected to HH received 0.9% NaCl injection directly after the exposure to hypoxia.

### 4.2. Immunohistochemistry

The animals (5–6 in each group) were anesthetized with 60 mg/kg of body weight of zoletil (Virbac) and 10 mg/kg of body weight of 2% xylazine (Interchemie). Then, transcardial perfusion was performed first with 100 mL of 0.9% NaCl followed by 100 mL of 4% paraformaldehyde in phosphate-buffered saline, pH 7,5. Quickly after that, the brain was removed, and the tissue block containing the hippocampus and the fronto-parietal neocortex was dissected. The tissue samples were postfixed overnight in 4% paraformaldehyde at +4 °C. The histological processing and immunohistochemical staining were performed as described in [55]. The reagents used were primary antibodies: rabbit anti-LC3 (Sigma-Aldrich Inc., St. Louis, MO, USA, SAB1306611) and rabbit anti-p62 (Sigma-Aldrich Inc., St. Louis, USA, P0067); the secondary biotinylated antibodies and avidin–biotin complex from the ABC-Peroxidase Kit (Vector Laboratories Inc., Burlingame, CA, USA, PK-4001); the secondary antibodies were anti-rabbit CF64 (Sigma-Aldrich Inc., St. Louis, USA, SAB4600352); DAPI (Sigma-Aldrich Inc., St. Louis, USA, D9542); DAB (Vector Laboratories Inc, USA, SK4100). The immunoperoxidase-stained slides were analyzed with an Olympus CX31 light microscope (Olympus, Tokyo, Japan) equipped with a Progress CT1 digital camera (Jenoptic, Germany). The images were processed using the ImageJ software (https://imagej.net/, accessed on 1 October 2020, RRID: SCR_003070) with the SDA plugin applied [56]. The mean optical density of the immunopositive cells was calculated. Primarily, the soma and the proximal part of the apical dendrite of the CA1 neurons of the hippocampus and neurons of the V layer of the neocortex were selected for analysis. The immunofluorescent-stained samples were visualized using the EVOS M5000 Imaging System (ThermoFisher Scientific, Waltham, MA, USA). The immunofluorescent images were used for comparative assessment of the LC3 and p62 staining and cell localization in control rats and rats subjected to hypoxia.

### 4.3. Protein Extraction and RNA Isolation Procedures

Animals from each group were sacrificed (5–6 animals in each), and the brains were immediately dissected on ice. The hippocampus and the frontal–parietal cortex from the left hemisphere were used for protein extraction, while those from the right hemisphere were used for RNA extraction.

Total proteins were extracted using the Laemmli sample preparation protocol. Shortly, the tissue extracts were homogenized in RIPA buffer containing protease inhibitor cocktail (Sigma Aldrich, P8340) and left for incubation for 30 min at +4 °C. The samples were centrifuged at 15,000× *g* for 20 min. The supernatant was aspirated and transferred into a clean tube. Then, the supernatant was diluted with the Laemmli sample buffer (0.5 of the supernatant volume) and put to boil for 5 min. After that, the samples were put on ice and stored at −20 °C before the analysis.

Total RNA from rat neocortex and hippocampus was isolated using Extact RNA reagent (Evrogen, Moscow, Russia, BC032) according to the manufacturer’s instruction. The isolated RNA concentration was measured on a Shimadzu UV mini-1240 spectrophotometer. The RNA quality was assessed using agarose gel electrophoresis. RNA was reversely transcribed into cDNA using the MMLV RT kit (Evrogen, Moscow, Russia, SK022S) and oligo-dT primer (Evrogen, Moscow, Russia, SB001, PB006).

### 4.4. Western Blot and Dot Analysis

**Figure A1:**
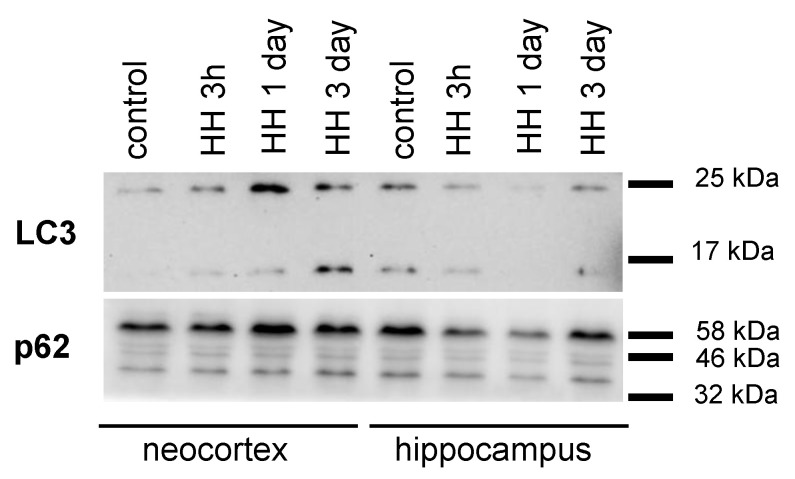
The representative immunoblots of LC3 and p62 in the hippocampus and neocortex of rats exposed to HH.

For Western blotting analysis, p62 proteins were separated by electrophoresis in 10% and LC3 protein in 20% sodium dodecyl sulfate-polyacrylamide gel (SDS-PAGE) and transferred to a PVDF membrane (0.2 μm, Thermo Scientific). The color pre-stained protein standard (New England BioLabs, Ipswich, MA, USA, P7712S) was used as a marker of the protein molecular weight. The dot blot method was performed according to the Abcam protocol (Abcam, Cambridge, UK). For dot analysis, 1 μL of the sample was immobilized on a nitrocellulose membrane (Protran 0.45 μm, Whatman). Both for Western blotting and dot analysis, the steps of the immunodetection were identical. For blocking of the nonspecific binding, the membranes were incubated in 5% milk for 1 h. Furthermore, the membranes were incubated with primary antibodies for 2 h at room temperature. Then, the membranes were washed with TBST and incubated with secondary HRP-anti-rabbit antibodies (Sigma Aldrich, A0545) for 1 h at room temperature. All steps of incubation were performed at continuous orbital shaking. Visualization was performed using an ECL detection kit (BioRad, 1705060) according to the manufacturer’s instructions. The protein bands and dots were visualized using the ChemiDoc MP Imaging System (Bio-Rad, Hercules, CA, USA). The dots were further counterstained with amido-black. The blots and dots were optically processed using the ImageJ software (https://imagej.net/, accessed on 1 October 2020). The integral optical density of each dot was calculated and then was normalized to the amount of total protein in the samples. The antibodies were verified on Western blotting, and the bands that the antibody recognized are presented in Figure A1.

### 4.5. qRT-PCR

qRT-PCR was performed in a StepOne Plus thermocycler (ABI) using qPCRmix-HS SYBR (Evrogen, PK147L). The primers for *map*/*lc3*, *tfeb* and *rpl13* were designed using Primer-BLAST (NCBI, http://www.ncbi.nlm.nih.gov/tools/primer-blast/, accessed on 1 October 2020; RRID: SCR_003095) and purchased from Evrogen. *tfeb*: forward—ACCAGAGGTAGAAATGGGAGC, reverse—CAAGGGAGCCAGAGGACACTA. *map*/*lc3*: forward—TCTTGGACGCTTGTACGCAT, reverse—TTGTGTGTCTCCTCAACCC. *rpl13*: forward—GAGCCCCAAGCCGCATTTTT, reverse—ACGCCCCAGGTAAGCAAACTT. The gene expression was calculated using the ΔΔCt method of relative quantification. The gene expression levels were normalized to the *rpl13* gene.

### 4.6. Statistics

The data were analyzed using the Microsoft Excel and Statistica Software 7 (Stat Soft, Inc., USA, http://www.statsoft.com/Products/STATISTICA/Product-Index, accessed on 1 October 2020; RRID: SCR_014213). The results are presented as the mean ± standard error of the mean (SEM). The Kruskal–Wallis test with Tukey–HSD analysis was used for comparison of the independent groups for immunohistochemical and dot analysis. In case of qRT-PCR analysis, statistical differences were assessed using the Kruskal–Wallis test and Mann–Whitney U test. The difference between the groups was considered significant if *p* < 0.05.

## 5. Conclusions

Taken together, in this work, we have indicated for the first time the important differences in the LC3 and p62 overall staining and puncta staining in the neurons of the hippocampus and neocortex in an animal model. The data obtained indicate that neurons of the CA1 field of the hippocampus and the V layer of the neocortex differ in the autophagic process under normoxic conditions. This feature is likely to underlie the difference between these brain regions in autophagic response to hypoxia. In the hippocampus, HH led to a decrease in LC3 and p62 levels with a consequent activation of related gene expression, whereas in the neocortex, the activation of autophagy was not pronounced and not associated with the changes in the protein or the mRNA levels. The observed differences between the hippocampus and neocortex in autophagy activity could possibly arise from the different ratio of the membrane receptors, which to a great extent determine the functional features of the neurons of these brain regions. The data obtained with the help of the HH model might be helpful for further investigation of the strategies by which neurons of different brain regions cope with the hypoxic stress of functional overload.

## Data Availability

The data presented in this study are available on request from the corresponding author.

## References

[1] Klionsky D.J., Emr S.D. (2000). Autophagy as a regulated pathway of cellular degradation. Science.

[2] Mizushima N., Komatsu M. (2011). Autophagy: Renovation of cells and tissues. Cell.

[3] Yu L., Chen Y., Tooze S.A. (2018). Autophagy pathway: Cellular and molecular mechanisms. Autophagy.

[4] Stavoe A.K.H., Holzbaur E.L.F. (2019). Autophagy in neurons. Annu. Rev. Cell Dev. Biol..

[5] Yamamoto A., Yue Z. (2014). Autophagy and its normal and pathogenic states in the brain. Annu. Rev. Neurosci..

[6] Hara T., Nakamura K., Matsui M., Yamamoto A., Nakahara Y., Suzuki-Migishima R., Yokoyama M., Mishima K., Saito I., Okano H. (2006). Suppression of basal autophagy in neural cells causes neurodegenerative disease in mice. Nature.

[7] Komatsu M., Waguri S., Chiba T., Murata S., Iwata J.-I., Tanida I., Ueno T., Koike M., Uchiyama Y., Kominami E. (2006). Loss of autophagy in the central nervous system causes neurodegeneration in mice. Nature.

[8] Nishiyama J., Miura E., Mizushima N., Watanabe M., Yuzaki M. (2007). Aberrant membranes and double-membrane structures accumulate in the axons of *Atg5*-null Purkinje cells before neuronal death. Autophagy.

[9] Boland B., Yu W.H., Corti O., Mollereau B., Henriques A., Bezard E., Pastores G.M., Rubinsztein D.C., Nixon R.A., Duchen M.R. (2018). Promoting the clearance of neurotoxic proteins in neurodegenerative disorders of ageing. Nat. Rev. Drug Discov..

[10] Chu C.T. (2019). Mechanisms of selective autophagy and mitophagy: Implications for neurodegenerative diseases. Neurobiol. Dis..

[11] Frake R.A., Ricketts T., Menzies F.M., Rubinsztein D.C. (2015). Autophagy and neurodegeneration. J. Clin. Investig..

[12] Ghavami S., Shojaei S., Yeganeh B., Andee S.R., Jangamreddy J.R., Mehrpour M., Christoffersson J., Chaabane W., Moghadam A.R., Kashani H.H. (2014). Autophagy and apoptosis dysfunction in neurodegenerative disorders. Prog. Neurobiol..

[13] Kim B.H., Jeziorek M., Kanal H.D., Contu V.R., Dobrowolski R., Levison S.W. (2021). Moderately inducing autophagy reduces tertiary brain injury after perinatal hypoxia-ischemia. Cells.

[14] Dohi E., Tanaka S., Seki T., Miyagi T., Hide I., Takahashi T., Matsumoto M., Sakai N. (2012). Hypoxic stress activates chaperone-mediated autophagy and modulates neuronal cell survival. Neurochem. Int..

[15] Giordano S., Darley-Usmar V., Zhang J. (2013). Autophagy as an essential cellular antioxidant pathway in neurodegenerative disease. Redox Biol..

[16] Hubbi M.E., Hu H., Kshitiz, Ahmed I., Levchenko A., Semenza G.L. (2013). Chaperone-mediated autophagy targets hypoxia-inducible factor-1α (HIF-1α) for lysosomal degradation. J. Biol. Chem..

[17] Tooze S.A., Codogno P. (2011). Compartmentalized regulation of autophagy regulators: Fine-tuning AMBRA1 by Bcl-2. EMBO J..

[18] Adhami F., Liao G., Morozov Y.M., Schloemer A., Schmithorst V.J., Lorenz J.N., Dunn R.S., Vorhees C.V., Wills-Karp M., Degen J.L. (2006). Cerebral ischemia-hypoxia induces intravascular coagulation and autophagy. Am. J. Pathol..

[19] Koike M., Shibata M., Tadakoshi M., Gotoh K., Komatsu M., Waguri S., Kawahara N., Kuida K., Nagata S., Kominami E. (2008). Inhibition of Autophagy Prevents Hippocampal Pyramidal Neuron Death after Hypoxic-Ischemic Injury. Am. J. Pathol..

[20] Puyal J., Clarke P.G. (2009). Targeting autophagy to prevent neonatal stroke damage. Autophagy.

[21] Rami A., Langhagen A., Steiger S. (2008). Focal cerebral ischemia induces upregulation of Beclin 1 and autophagy-like cell death. Neurobiol. Dis..

[22] Rybnikova E., Vataeva L., Tyulkova E., Gluschenko T., Otellin V., Pelto-Huikko M., Samoilov M.O. (2005). Mild hypoxia preconditioning prevents impairment of passive avoidance learning and suppression of brain NGFI-A expression induced by severe hypoxia. Behav. Brain Res..

[23] Rybnikova E., Sitnik N., Gluschenko T., Tyulkova E., Samoilov M.O. (2006). The preconditioning modified neuronal expression of apoptosis-related proteins of Bcl-2 superfamily following severe hypobaric hypoxia in rats. Brain Res..

[24] Rybnikova E., Nalivaeva N. (2021). Glucocorticoid-dependent mechanisms of brain tolerance to hypoxia. Int. J. Mol. Sci..

[25] Rybnikova E., Glushchenko T., Churilova A., Pivina S., Samoilov M. (2011). Expression of glucocorticoid and mineralocorticoid receptors in hippocampus of rats exposed to various modes of hypobaric hypoxia: Putative role in hypoxic preconditioning. Brain Res..

[26] Stroev S.A., Gluschenko T.S., Tjulkova E.I., Rybnikova E.A., Samoilov M.O., Pelto-Huikko M. (2005). The effect of preconditioning on the Cu, Zn superoxide dismutase expression and enzyme activity in rat brain at the early period after severe hypobaric hypoxia. Neurosci. Res..

[27] Samoilov M., Churilova A., Gluschenko T., Rybnikova E. (2014). Neocortical pCREB and BDNF expression under different modes of hypobaric hypoxia: Role in brain hypoxic tolerance in rats. Acta Histochem..

[28] Boland B., Kumar A., Lee S., Platt F.M., Wegiel J., Yu W.H., Nixon R.A., Boland B., Kumar A., Lee S. (2008). Autophagy Induction and Autophagosome Clearance in Neurons: Relationship to Autophagic Pathology in Alzheimer’s Disease. J. Neurosci..

[29] Klionsky D.J., Abdel-Aziz A.K., Abdelfatah S., Abdellatif M., Abdoli A., Abel S., Abeliovich H., Abildgaard M.H., Abudu Y.P., Acevedo-Arozena A. (2021). Guidelines for the use and interpretation of assays for monitoring autophagy (4th edition). Autophagy.

[30] Mizushima N., Yoshimori T. (2007). How to Interpret LC3 Immunoblotting. Autophagy.

[31] Vodicka P., Lim J., Williams D.T., Kegel K.B., Chase K., Park H., Marchionini D., Wilkinson S., Mead T., Birch H. (2014). Assessment of chloroquine treatment for modulating autophagy flux in brain of WT and HD mice. J. Huntington’s Dis..

[32] Mizushima N., Yoshimori T., Levine B. (2010). Methods in mammalian autophagy research. Cell.

[33] Hill S., Colón-Ramos D.A. (2020). The journey of the synaptic autophagosome: A cell biological perspective. Neuron.

[34] Birdsall V., Waites C.L. (2019). Autophagy at the synapse. Neurosci. Lett..

[35] Kuijpers M., Kochlamazashvili G., Stumpf A., Puchkov D., Swaminathan A., Lucht M.T., Krause E., Maritzen T., Schmitz D., Haucke V. (2021). Neuronal autophagy regulates presynaptic neurotransmission by controlling the axonal endoplasmic reticulum. Neuron.

[36] Lieberman O.J., Sulzer D. (2020). The synaptic autophagy cycle. J. Mol. Biol..

[37] Shehata M., Abdou K., Choko K., Matsuo M., Nishizono H., Inokuchi K. (2018). Autophagy enhances memory erasure through synaptic destabilization. J. Neurosci..

[38] Shen W., Ganetzky B. (2009). Autophagy promotes synapse development in *Drosophila*. J. Cell Biol..

[39] Stavoe A.K., Hill S.E., Hall D.H., Colón-Ramos D.A. (2016). KIF1A/UNC-104 transports ATG-9 to regulate neurodevelopment and autophagy at synapses. Dev. Cell.

[40] Nikoletopoulou V., Sidiropoulou K., Kallergi E., Dalezios Y., Tavernarakis N. (2017). Modulation of autophagy by BDNF underlies synaptic plasticity. Cell Metab..

[41] Spruston N. (2008). Pyramidal neurons: Dendritic structure and synaptic integration. Nat. Rev. Neurosci..

[42] Kessels H.W., Malinow R. (2009). Synaptic AMPA receptor plasticity and behavior. Neuron.

[43] Cotman C.W., Monaghan D.T. (1986). Anatomical organization of excitatory amino acid receptors and their properties. Adv. Exp. Med..

[44] Shehata M., Matsumura H., Okubo-Suzuki R., Ohkawa N., Inokuchi K. (2012). Neuronal stimulation induces autophagy in hippocampal neurons that is involved in AMPA receptor degradation after chemical long-term depression. J. Neurosci..

[45] Glatigny M., Moriceau S., Rivagorda M., Ramos-Brossier M., Nascimbeni A.C., Lante F., Shanley M.R., Boudarene N., Rousseaud A., Friedman A.K. (2019). Autophagy is required for memory formation and reverses age-related memory decline. Curr. Biol..

[46] Niu M., Zheng N., Wang Z., Gao Y., Luo X., Chen Z., Fu X., Wang Y., Wang T., Liu M. (2020). RAB39B Deficiency impairs learning and memory partially through compromising autophagy. Front. Cell Dev. Biol..

[47] Vetrovoy O., Sarieva K., Galkina O., Eschenko N., Lyanguzov A., Gluschenko T., Tyulkova E., Rybnikova E. (2019). Neuroprotective mechanism of hypoxic post-conditioning involves HIF1-associated regulation of the pentose phosphate pathway in rat brain. Neurochem. Res..

[48] Tremblay R., Lee S., Rudy B. (2016). GABAergic interneurons in the neocortex: From cellular properties to circuits. Neuron.

[49] Schaaf M.B.E., Keulers T.G., Vooijs M.A., Rouschop K.M.A. (2016). LC3/GABARAP family proteins: Autophagy-(un)related functions. FASEB J..

[50] Hui K.K., Takashima N., Watanabe A., Chater T.E., Matsukawa H., Nekooki-Machida Y., Nilsson P., Endo R., Goda Y., Saido T.C. (2019). GABARAPs dysfunction by autophagy deficiency in adolescent brain impairs GABA_A_ receptor trafficking and social behavior. Sci. Adv..

[51] Kim J.K., Kim Y.S., Lee H.-M., Jin H.S., Neupane C., Kim S., Lee S.-H., Min J.-J., Sasai M., Jeong J.-H. (2018). GABAergic signaling linked to autophagy enhances host protection against intracellular bacterial infections. Nat. Commun..

[52] Lakhani R., Vogel K.R., Till A., Liu J., Burnett S.F., Gibson K.M., Subramani S. (2014). Defects in GABA metabolism affect selective autophagy pathways and are alleviated by m TOR inhibition. EMBO Mol. Med..

[53] Luscher B., Fuchs T., Kilpatrick C.L. (2011). GABAA receptor trafficking-mediated plasticity of inhibitory synapses. Neuron.

[54] Du Sert N.P., Hurst V., Ahluwalia A., Alam S., Avey M.T., Baker M., Browne W.J., Clark A., Cuthill I.C., Dirnagl U. (2020). The ARRIVE guidelines 2.0: Updated guidelines for reporting animal research. PLoS Biol..

[55] Churilova A.V., Gluschenko T.S., Rybnikova E.A., Samoilov M.O. (2019). The effect of histone deacetylase inhibitor on the expression level of glucococrticoid receptor in rat forebrain under hypoxia. Cell Tissue Biol..

[56] Nurzynska K., Mikhalkin A., Piorkowski A. (2017). CAS: Cell annotation software—Research on neuronal tissue has never been so transparent. Neuroinformatics.

